# Epidemiology of classic psychedelic substances: results from a Norwegian internet convenience sample

**DOI:** 10.3389/fpsyt.2023.1287196

**Published:** 2023-11-13

**Authors:** Tor-Morten Kvam, Malin V. Uthaug, Kristoffer A. A. Andersen, Birk Berggrav Refsum, Paula Aarseth Tunstad, Lowan Han Stewart, Henrik Børsting Jacobsen, Cato Grønnerød

**Affiliations:** ^1^Faculty of Medicine, University of Oslo, Oslo, Norway; ^2^Nordre Østfold DPS, Østfold Hospital Trust, Grålum, Norway; ^3^The Centre for Psychedelic Research, Department of Brain Sciences, Faculty of Medicine, Imperial College London, London, United Kingdom; ^4^Somnivore Pty. Ltd., Bacchus Marsh, VIC, Australia; ^5^Department of Neuropsychology and Psychopharmacology, Faculty of Psychology and Neuroscience, Maastricht University, Maastricht, Netherlands; ^6^Psykolyse, Oslo, Norway; ^7^Department of Psychology, University of Oslo, Oslo, Norway; ^8^Department of Pain Management and Research, Oslo University Hospital, Oslo, Norway

**Keywords:** psychedelics, psilocybin, lysergic acid diethylamide (LSD), *N,N*-dimethyltryptamine (DMT), survey, mental disorders, substance use disorders

## Abstract

**Objective:**

In recent years, there has been a renewed interest in investigating the use of classic psychedelics such as psilocybin and lysergic acid diethylamide (LSD) in the treatment of mental disorders and substance use disorders. However, knowledge about the epidemiology of classic psychedelics in the Nordic countries is limited.

**Methods:**

We recruited adult, Norwegian participants who have had a memorable experience after taking a classic psychedelic substance. They filled in an anonymous internet survey with 119 items covering matters related to recreational use of psychedelics using a secure, web-based application. Data are presented by using descriptive statistics (frequencies, means, and standard deviations).

**Results:**

We recruited 841 participants, 770 (72% male; 88% 45 years or younger) of which were included in the data analysis. The intentions behind taking the psychedelic substance were mainly recreational (46.1%) or therapeutic (42.3%). Most participants reported that their most memorable experience was with psilocybin. As in modern era clinical trials, most participants were well-prepared before, did processing during, and did integration work after the experience, whereas only a minority were supported by a therapist. Self-perceived symptoms of various mental disorders and substance use disorders were prevalent in the sample. Most subjects reported improvements in their condition. Although adverse reactions were usually mild and short-lived, 4.2% lasted for 1 year or more. Persisting flashbacks were present for a year or more among 2.9% of the participants.

**Conclusion:**

In this cross-sectional sample of Norwegian, self-selecting adults, we shed light on what characterizes the most memorable experience with a classic psychedelic substance, including short- and long-term risks and benefits. For the most part, the psychedelic experience led to improvements in self-perceived symptoms of mental disorders and substance use disorders. However, a small subset experienced persisting adverse reactions.

## Introduction

A renewed interest has recently surged in investigating the therapeutic use of psychedelic substances. Modern studies with psilocybin have shown promising results in the treatment of anxiety and depression related to a life-threatening illness (LTI) (1, 2), major depressive disorder (3, 4), treatment-resistant depression (5), and alcohol use disorder (AUD) (6), whereas one study found LSD effective for anxiety with or without LTI (7).

Classic psychedelic substances include the synthetic substance LSD and the naturally occurring substances psilocybin from various mushroom species, mescaline from peyote cactus, and N,N-dimethyltryptamine (DMT) from the brew ayahuasca (8). These substances exert their effect primarily through stimulation of the serotonin (5HT) 2A receptor (8). Naturally occurring psychedelic substances have probably been used for thousands of years for spiritual, religious and medicinal purposes (8).

After decades of severe restrictions on research due to an international ban, there has been a huge increase in research activity worldwide in recent years, and promising results in trials with rigorous design warrant investigation in confirmatory, phase 3 trials.

Even though classic psychedelic substances are generally considered physiologically safe with a low toxicity, the psychedelic experience is often psychologically challenging, and individuals may put themselves at risk during the acute experience (8). A review of both the clinical (including emergency department case reports) and scientific literature found the following acute side effects of classic psychedelic substances: terrifying illusions, anxiety rising to panic and paranoia, confusion, aggression, depression with increased risk of suicide (9). The side effects rarely lasted beyond 48 h, however. Hallucinogen persisting perception disorder (HPPD) is a condition with persisting side effects characterized by sustained, distressing flashbacks long after acute exposure to a psychedelic substance. HPPD is considered a rare consequence of psychedelic use, but more knowledge is needed about its prevalence (10).

Epidemiological studies from recreational use of psychedelic substances supplement findings from clinical studies. Large population studies with over one hundred thousand participants have shown that previous use of psychedelic substances is associated with a reduced risk of mental disorders, suicide, and antisociality compared to the general population (11, 12). Although with inherent limitations, empirical evidence from internet surveys can contribute to increased knowledge about harms and benefits from psychedelic experiences. A retrospective internet survey of almost two thousand participants that aimed to map the most challenging experience after consuming psilocybin mushrooms found that 39% ranked the experience among the five most challenging experiences of their lives. Additionally, 11% had put themselves or others in a risky situation, but physical and social support reduced the risk (13). A prospective internet survey examined participants before and after a planned psychedelic experience to study which factors could predict change in well-being (14). The authors found that baseline personality traits were the strongest predictors for well-being after the psychedelic experience. A retrospective internet survey following the use of the psychedelic substance 5-methoxy-*N,N*-dimethyltryptamine (5-MeO-DMT) found that 37% of the participants had challenging experiences (15). A small subset (4–7%) experienced worsening of mental health conditions such as anxiety, depression and substance abuse, whereas the majority experienced improvements of various conditions such as depression (77%), post-traumatic stress disorder (PTSD; 79%), anxiety (69%), substance abuse (63%), and obsessive-compulsive disorder (OCD; 53%). Another retrospective internet survey with 452 participants reporting from their most memorable experience with the classic psychedelic substance mescaline found that the motivations for use were the exploration of spirituality and connection with nature (16). About half of the participants reported a history of a mental disorder and two thirds reported improvements in their condition due to the mescaline experience. Nine percent reported cravings following intake of mescaline and only 1 % reported legal problems. About one quarter to one third rated the experience as one of the top five or single most personally meaningful, spiritually significant, or psychologically insightful experiences, while 11% rated the experience as one of the top five or single most psychologically challenging experiences. In an internet survey exploring the encounter of entities during the most memorable experience with DMT, about half of the 2,500 participants rated the experience as one of the top five or single most personally meaningful, spiritually significant, or psychologically insightful experiences, while about one third rated the experience as one of the top five or single most psychologically challenging experiences (17). Almost the exact same figures regarding the most memorable psychedelic experience in relation to other life events were found in a Danish internet survey exploring the use of classic psychedelics (18). In this survey, 500 participants reported that the motivations for use were mainly curiosity as well as spiritual and therapeutic purposes. 29% of the participants reported a history of mental disorder. There was a self-perceived improvement in 81% across all mental disorders, including an 87% improvement in both anxiety and depression, and a 90% improvement of PTSD. Overall, respondents in various internet surveys report that psychedelics can lead to highly challenging experiences, but they are very often also reported as highly memorable and even life changing.

Although similar surveys as the present study have been previously conducted in English-speaking countries, we know less about the epidemiology of classic psychedelics in the Nordic countries, and surveys in non-English speaking countries have been encouraged (15). A nationwide cross-sectional survey (N > 50,000) of Norwegian students aged 18–35 found 2.1% use of psilocybin and/or LSD the past 12 months (19). In a previous study by the same researchers, 8% reported lifetime use of psilocybin in a representative sample of the Norwegian population (20). But beyond the frequency of use, however, more knowledge is needed about the acute and persisting effects from experience with a classic psychedelic substance, what characterizes such an experience, and the impact on self-perceived mental disorders and substance use disorders in a Norwegian population. This is especially important due to anecdotal reports of psychedelic substance use and abuse amongst the general population in Norway. Less is known, however, about the extent of recreational use of drugs, and psychedelics in particular, and the effects users experience from such use. The current paper will shed some light on this issue.

The primary aim of the present study was to map the epidemiology of classic psychedelic substance use in a Norwegian population. Specifically, among those who are willing to report such use, we wanted to study characteristics of, and acute and persisting effects from, the most memorable experience with a classic psychedelic substance. Furthermore, we wanted to shed light on what factors contributed to someone’s most memorable experience.

The sub-aims of the study were (1) to study subjectively experienced changes in symptoms of mental disorders and substance use disorders after a psychedelic experience, and (2) to study the occurrence, duration and severity of persisting adverse reactions, including questions exploring persistent perceptual disturbances as a proxy for HPPD.

## Methods

### Recruitment and respondent characteristics

We conducted an anonymous online survey containing a total number of 119 items. Data collections were handled by questionnaires created with Nettskjema.no, a survey solution developed and hosted by the University of Oslo. To take part in the study, the participants had to confirm to be (1) 18 years of age or older, (2) able to read, write and speak fluent Norwegian, and (3) have had a memorable experience after taking a classic psychedelic substance (LSD, psilocybin, 2C-B, ayahuasca, DMT, 5-MeO-DMT, mescaline, or others). Psychedelic experiences from ketamine and methylenedioxymethamphetamine (MDMA) were omitted from this survey. Participants were recruited via the web pages and social media groups of the Norwegian Association for Psychedelic Science (21), Rusopplysningen.no (an information service about commonly used drugs), and numerous Facebook-pages for people with an interest in psychedelics.

Participation in the study was voluntary and anonymous, and participants were not compensated in any way. By starting the survey, the participants consented to take part in the study. We only analyzed data from survey completers (i.e., those who completed the survey in full). The participants were informed that even after they started the survey, they could cancel at any time, and if so, none of their answers will be used in the study.

The participants were asked about gender, age, education level, annual income group, and marital status. Subjects provided information on how many times they had used classic psychedelic substances previously in their lifetime, and how many times in the past year.

### Characteristics of the most memorable experience

The participants answered the following questions about the most memorable experience: which substance administered, time since administration, to which degree they remembered the experience, the setting in which the psychedelic substance was taken, any support during the psychedelic experience, motivation for taking the psychedelic substance, collaboration with therapist, guide or trip-sitter if any, dosage, preparation, intent, wish for change, co-administration of alcohol or substances, and integration after the psychedelic experience. To uncover any biases in the sample related to the participants’ positive expectations of psychedelic substances, the participants were asked to which degree they agreed that psychedelic substances should be used therapeutically.

### Acute and persisting effects

The subjects were given the opportunity to answer questions about any increased understanding of the significance of past life events during the psychedelic experience, to what extent they uncovered any associations between current and past interpersonal relationships during the psychedelic experience, and to what extent they discovered any new or forgotten ways of dealing with difficulties and challenges during the psychedelic experience.

We also asked the participants about the occurrence of persisting adverse reactions, and the severity and duration of such events after the psychedelic experience, and if they sought professional help to handle adverse reactions. We asked specifically about flashbacks and any functional impairment related to the flashbacks. We also asked about craving symptoms and failed attempts to resist new intake of psychedelic substances.

As in former studies, e.g., (22), we asked the participants to compare their most memorable experience to other meaningful experiences in life, i.e., how meaningful, spiritual, psychologically challenging, and insightful the psychedelic experience was. Furthermore, we asked about subjectively perceived persisting changes in personal well-being/life-satisfaction, purpose and meaning of life, social relationships, mood, behavior, spirituality, as well as attitudes about life, nature, and death.

### Questionnaires

We also included three standardized questionnaires in the survey, the Ego-Dissolution Inventory (EDI), the Emotional Breakthrough Inventory (EBI), and the Challenging Experience Questionnaire (CEQ). All three were translated, back-translated, pilot tested and revised to Norwegian by one of the co-authors (Kristoffer A. A. Andersen, masked for review). Psychometric validation papers for all three questionnaires are currently underway, but have not yet been published. However, the most recent versions as of august 2023 are included in this survey.

EDI is an eight-item self-report questionnaire that measures the level of ego-dissolution in a psychedelic experience (23). The EDI consists of eight statements to be assessed on a scale from 0 (“no, not more than usually”) to 100 (“yes, entirely or completely”). A sample statement is “I experienced a dissolution of my “self” or “ego.”

EBI aims to map any emotional breakthrough as a distinct part of a psychedelic experience (24). The EBI consists of six statements on a scale from 0 (“no, not more than usually”) to 100 (“yes, entirely or completely”). An example of a statement is “I faced emotionally difficult feelings that I usually push aside.”

CEQ is a 26-item self-report questionnaire that measures the perceived challenging aspects of a psychedelic experience (25). The participants were asked to rate their most memorable experience with a classic psychedelic substance on a five-point scale from 0 (“none: not at all”) to 5 (“extreme”). The CEQ is divided into seven dimensions: (1) Isolation, (2) Grief, (3) Physical distress, (4) Fear, (5) Insanity, (6) Paranoia, and (7) Death. A sample statement is “Isolation and loneliness.”

### Mental disorders and substance use disorders

We asked the participants if they had been diagnosed with or suspected that they would meet the criteria for the following mental disorders: social anxiety, panic disorder, OCD, depression, bipolar disorder, psychosis/schizophrenia, eating disorder, PTSD, attention deficit hyperactivity disorder (ADHD), autism spectrum disorder (ASD), and suicidality. Furthermore, we asked about substance use disorders, including nicotine dependence (daily smoking), and abuse of or addiction to alcohol or other substances. The latter included illegal drugs other than psychedelics, and addictive prescription drugs.

### Ethical considerations and data protection

We stated in the information sheet that this survey should not be seen as a defense of psychedelic substances or a call for the use of psychedelic substances. We collected research data from people who have already used psychedelic substances. We sent a request to the regional ethics committee (reference number 212268), but the study was considered not to be required to be submitted to the committee for external evaluation, as we only collected anonymous data. We did not collect identifiable information such as name, email address or IP address. The study was approved by the Internal ethics committee at the Department of Psychology, University of Oslo (reference number 18183844). We used a secure web application for data storage (Services for Sensitive Data; TSD), which meets requirements for the processing and storage of sensitive research data.

Prior to the start of data collection, the survey was reviewed by a focus group of five volunteers recruited from the Norwegian Association for Psychedelic Science as well as a designated user representative at Østfold Hospital Trust. Their task was to flag problematic, incomprehensible or ambiguous questions. As a result of feedback from the focus group, we changed the wording of some questions, but no questions were added or removed.

### Statistical analysis

We present descriptive statistics for demographics, previous use of classic psychedelic substances, lifetime history of mental disorders and substance use disorders, characteristics of the most memorable psychedelic experience, and persisting adverse reactions and benefits from the experience. We used a t-test to look for differences in the frequency of psychedelic use between those with or without mental disorders and substance use disorders. Analyses were run using IBM SPSS.

## Results

### Respondent characteristics

**Figure 1:**
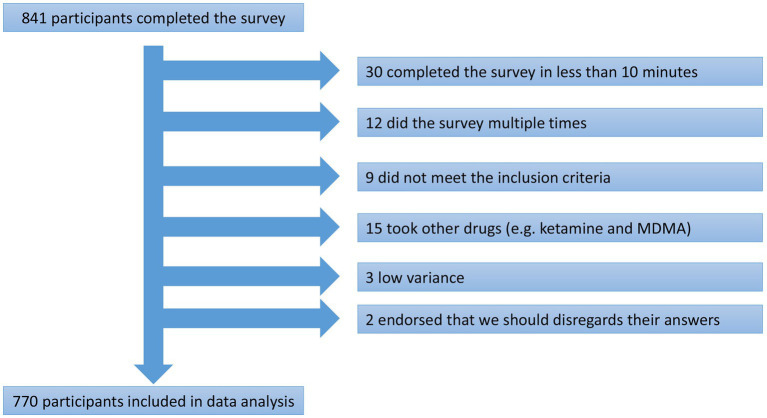
Reasons for excluding 71 participants from the data analysis.

Data collection took place between May 9th and August 19th, 2022. Of 841 participants who completed the survey, 770 were deemed eligible for the data analysis. See Figure 1 for details about reasons for excluding 71 participants. Of the 770 included in the data analysis, 555 were male (72%), 208 female (27%) and seven were non-binary (1%, see Table 1 for demographics). The majority of participants were 45 years old or younger (88%) and 63% reported some degree of higher education. The annual income was heterogeneous, and 86.6% of the participants were unmarried.

**Table 1:** Demographics.

**Gender**	
Female	208 (27.0%)
Male	555 (72.1%)
Non-binary	7 (0.9%)
**Age**	
18–25 years	194 (25.2%)
26–35 years	315 (40.9%)
36–45 years	170 (22.1%)
46–55 years	62 (8.1%)
56–65 years	23 (3.0%)
66+ years	6 (0.8%)
**Education**	
Not completed primary school^(1)^	63 (8.2%)
Not completed upper secondary school^(2)^	3 (0.4%)
Completed upper secondary school	222 (28.8%)
Completed vocational school^(3)^	93 (12.1%)
Completed higher education (university/college) up to 4 years	173 (22.5%)
Completed higher education (university/college) more than 4 years	216 (28.1%)
**Annual income (NOK)**	
Less than 300,000	144 (18.7%)
300,000–399,000	69 (9.0%)
400,000–499,000	75 (9.7%)
500,000–749,000	176 (22.9%)
750,000–999,999	118 (15.3%)
1,000,000–1,249,999	90 (11.7%)
1,250,000–1,499,999	53 (6.9%)
1 500,000 or more	45 (5.8%)
**Relationship status**	
Married	103 (13.4%)
Cohabitant	261 (33.9%)
Divorced or separated	46 (6%)
Widower/widow	5 (0.6%)
Single	355 (46.1%)
**Employment status**	
Working full time	457 (59.4%)
Working part-time	72 (9.4%)
Unemployed	18 (2.3%)
Disability benefit	86 (4.2%)
Pensioner	4 (0.5%)
Pupil/student	133 (17.3%)

^(1)^10 years, ^(2)^3 years, and ^(3)^4 years.

The median response time was 20 min and 26 s. As it was possible to finish the survey in more than one session, the response time distribution was right skewed with a mean of 57.93 min (SD = 223.25).

Table 2 shows reported previous use of classic psychedelics. Only 51 participants (6.6%) reported only one experience with a psychedelic substance, whereas 49% of the participants reported the use of a psychedelic substance one to five times the past year, and 21% reported no use of a psychedelic substance the past year. The vast majority of the participants agreed that psychedelic substances should be used therapeutically: 74% answered that they strongly agreed and 21% that they agreed.

**Table 2:** Previous use of and attitudes toward therapeutic use of classic psychedelics.

**Life-time use of classic psychedelics**	
Just on this one occasion	51 (6.6%)
1–5	180 (23.4%)
5–10	176 (22.9%)
10–20	136 (17.7%)
20–50	132 (17.1%)
50+	95 (12.3%)
**Past year use of classic psychedelics**	
0	162 (21%)
1	85 (11%)
1–5	378 (49.1%)
5–10	96 (12.5%)
10–20	33 (4.3%)
20–50	13 (1.7%)
50+	3 (0.4%)
**Positive attitudes toward the therapeutic use of classic psychedelics**	
Strongly agree	570 (74%)
Agree	164 (21.3%)
Neither agree nor disagree	23 (3%)
Disagree	10 (1.3%)
Strongly disagree	3 (0.4%)

### Characteristics of the most memorable psychedelic experience

As shown in Table 3, most of the participants reported a memorable experience with psilocybin (53%), followed by LSD (33%), DMT (4.7%), and ayahuasca (4.4%). The majority of the participants considered the dose to be moderate (54%) or high (38%). The vast majority of participants reported that they remembered the experience quite clearly (41.6%) or very clearly (50.6%). The settings in which most participants used psychedelic substances were at home (33%), at somebody else’s home (21.9%) or outdoors/in nature (25.2%). 17.3% were alone during the experience with the classic psychedelic substance, whereas 39.9% received guidance or support from others. Of the 28.1% who stated collaboration with a therapist, guide or trip-sitter, most answered that the collaboration was very constructive (138/216 = 63.9%) or somewhat constructive (44/216 = 20.4%). Note that this proportion is different from the proportion of participants endorsed in which setting the psychedelic substance was taken (therapist’s clinic or with a guide/trip sitter; 3.1%). The reason for this discrepancy is not known to us.

**Table 3:** Characteristics of the most memorable experience with a classic psychedelic drug.

**Which classic psychedelic**	
Psilocybin	407 (52.9%)
LSD	254 (33%)
DMT	36 (4.7%)
Ayahuasca	34 (4.4%)
2C-B	21 (2.7%)
Mescaline	5 (0.6%)
5-MeO-DMT	2 (0.3%)
Others	11 (1.4%)
**Dose**	
Low	55 (7.1%)
Moderate	420 (54.5%)
High	295 (38.3%)
**How well remembered**	
Not so well	3 (0.4%)
Some	57 (7.4%)
Quite clear	320 (41.6%)
Very clear	390 (50.6%)
**Setting**	
At home	254 (33%)
At somebody else’s home	169 (21.9%)
Outdoors, in nature	194 (25.2%)
Outdoors, urban environment	22 (2.9%)
Church/other religious or ceremonial/spiritual setting	23 (3%)
At a festival	16 (2.1%)
Therapeutic clinic or with a guide/trip sitter	24 (3.1%)
Other	68 (8.8%)
**Support**	
Alone	133 (17.3%)
Not alone, but did not receive guidance or support	330 (42.9%)
Received guidance or support	307 (39.9%)
**Motivation for taking the psychedelic drug**	
Recreational, for fun or out of curiosity	355 (46.1%)
Therapeutic: desire for gained insight, reduction of symptoms, increased quality of life or processing of difficult memories or feelings	326 (42.3%)
Wish for a spiritual or religious experience	67 (8.7%)
Escape or distraction from discomfort, challenges in life, or boredom	22 (2.9%)
**Collaboration with a therapist, guide or trip-sitter**	
Very unconstructive	4 (0.5%)
Somewhat unconstructive	6 (0.8%)
Neither/or	24 (3.1%)
Somewhat constructive	44 (5.7%)
Very constructive	138 (17.9%)
Not applicable (did not take the psychedelic drug with a therapist)	554 (71.9%)
**Preparation**	
To a very small extent	40 (5.2%)
To a small extent	71 (9.2%)
Neither/or	111 (14.4%)
To a large extent	351 (45.6%)
To a very large extent	197 (25.6%)
**Intention**	
To a very small extent	43 (5.6%)
To a small extent	107 (13.9%)
Neither/or	215 (27.9%)
To a large extent	273 (35.5%)
To a very large extent	132 (17.1%)
**Desire for change**	
To a very small extent	66 (8.6%)
To a small extent	120 (15.6%)
Neither/or	302 (39.2%)
To a large extent	176 (22.9%)
To a very large extent	106 (13.8%)
**Integration**	
To a very small extent	43 (5.6%)
To a small extent	90 (11.7%)
Neither/or	146 (19%)
To a large extent	368 (46.8%)
To a very large extent	123 (16%)
**Co-administration of alcohol or illicit drugs**	
Yes	289 (37.5%)
No	456 (59.2%)
Uncertain	25 (3.2%)
**Substance co-administered**	
Dissociatives (Ketamine, PCP, etc.)	16 (2.1%)
Cannabis (hash, marihuana, THC)	233 (30.3%)
Central nervous system stimulants (amphetamine, methamphetamine, metylphenidate)	21 (2.7%)
Cocaine	18 (2.3%)
Alcohol	99 (2.9%)
Ecstasy/MDMA	36 (4.7%)
Opiates (morphine, heroin, methadone, codeine, opium)	5 (0.6%)
Volatile substances (glue, ethyl chloride, nitrous oxide, amyl nitrate or butyl nitrate)	4 (0.5%)
Anxiolytics (benzodiazepines, barbiturates, GHB)	10 (1.3%)
Others	
**Questionnaires [mean, (SD)]**	
EDI (scale from 0 to 800)	460.22 (199.76)
EBI (scale from 0 to 600)	338.19 (161.94)
CEQ total (scale from 0 to 130)	52.25 (24.12)
CEQ subscales	
Isolation (3 items; scale from 0 to 15)	6.09 (3.75)
Grief (6 items; scale from 0 to 30)	13.75 (7.42)
Physical distress (5 items; scale from 0 to 25)	9.62 (4.77)
Fear (5 items; scale from 0 to 25)	10.89 (6.45)
Insanity (3 items; scale from 0 to 15)	5.82 (4.08)
Paranoia (2 items; scale from 0 to 10)	2.74 (1.51)
Death (2 items; scale from 0 to 10)	3.34 (2.67)

The motivation for taking the psychedelic substance was mainly recreational/for fun or out of curiosity (46.1%), or therapeutic (wish for increased insight, symptom reduction, increased quality of life or processing of memories/emotions; 42.3%), while a smaller proportion stated a wish for a spiritual or religious experience (8.7%), or escape or distraction from discomfort, life challenges or boredom (2.9%). Most of the participants stated that they were prepared for the psychedelic experience – to a large extent (45.6%) or to a very large extent (25.6%). Most of the participants also endorsed taking the psychedelic substance with some level of intention – to a large extent (35.5%) or to a very large extent (17.1%). Furthermore, most of the participants stated that they had done integration work after the psychedelic experience to a large (46.8%) or very large extent (16.0%). Regarding the question about wish for change, the participants’ answers were more dispersed: 39.2% endorsed either/or, while 22.9 and 13.8% of the participants stated wish for change to a large extent or to a very large extent, respectively. 37.5% of the participants co-administered alcohol or other drugs with the psychedelic substance. The by far most commonly co-administered drug was cannabis (in 30.3% of the cases), followed by ecstasy/MDMA (4.7%) and alcohol (2.9%).

### Acute and persisting effects

95.1% of the participants reported increased understanding of the significance of past life events during the psychedelic experience. 94.2% reported that they uncovered associations between current and past interpersonal relationships during the psychedelic experience. 92.7% of the participants reported that they discovered new or forgotten ways of dealing with difficulties and challenges during the psychedelic experience.

The degree of ego dissolution as measured with the EDI (8 items) was on average 58 points per item on a scale from 0 to 100. Similarly, the degree of emotional breakthrough as measured with the EBI (6 items) was on average 56 points per item on a scale from 0 to 100, while challenging experiences as measured with the CEQ were on average 2.0 per item on a scale from 0 to 5. See Table 3 for details.

As shown in Table 4, compared to other life experiences on a scale from 1 to 8, the participants rated their most memorable experience with a classic psychedelic substance as personally meaningful, spiritual, and psychologically challenging, and moderately psychological insightful. Additionally, about one quarter of the participants (range 24–31%) rated the experience as among the top five or the single most personally meaningful, spiritual, or psychologically insightful experience. Fourteen percent rated it as among the top five or single most psychologically challenging experience. Table 4 also shows that the majority of the participants reported positive and desirable change that they attributed to the experience with the classic psychedelic substance, including well-being and life satisfaction (91%), life’s purpose (86%), life’s meaning (84%), social relationships (82%), attitudes about life (89%), attitudes about nature (80%), attitudes about death (60%), mood (77%), behavior (73%) and spirituality (66%).

**Table 4:** Comparison to other life experiences and change attributed to the experience.

**Comparison to other lifetime experiences [ratings from 1 to 8, mean, (SD)]**	
Personally meaningful	5.89 (1.50)
Spiritual	5.90 (2.17)
Psychologically insightful	4.04 (2.21)
Psychologically challenging	5.99 (1.68)
**Percentage rating the item as among the top 5 or single most of lifetime**	
Personally meaningful	235 (30.5%)
Spiritual	187 (24.3%)
Psychologically insightful	238 (30.9%)
Psychologically challenging	107 (13.9%)
**Percentage rating the item single most of lifetime**	
Personally meaningful	91 (11.8%)
Spiritual	223 (29%)
Psychologically insightful	136 (17.7%)
Psychologically challenging	39 (5.1%)
**Persisting changes from the most memorable psychedelic experience [ratings of change from 1 (most positive change) to 7 (most negative change), mean, (SD)]**	
Life satisfaction and general well-being	1.97 (1.11)
Social relationships	2.30 (1.16)
Meaning of life	2.25 (1.14)
Mood	2.56 (1.14)
**Percentage rating positive desirable change**	
Well-being and life satisfaction	90.8%
Life’s purpose	86%
Life’s meaning	83.5%
Social relationships	82.2%
Attitudes about life	88.8%
Attitudes about nature	79.9%
Attitudes about death	59.7%
Mood	76.5%
Behavior	72.7%
Spirituality	65.6%

### Perceived change of mental disorders and substance use disorders

**Figure 2:**
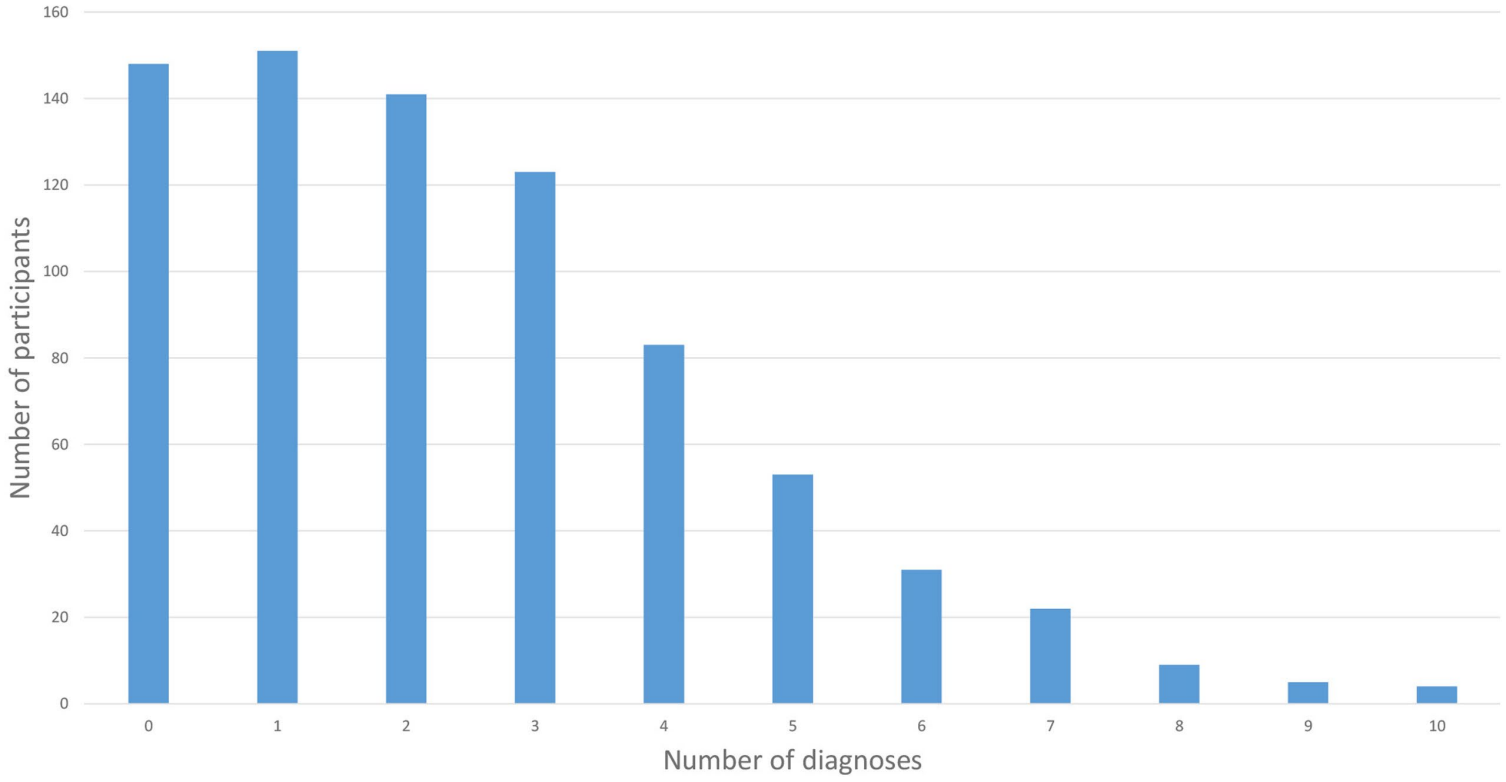
Number of diagnoses.

As shown in Table 5 – History of mental disorders and substance use disorders, the most prevalent self-perceived or clinician-assessed diagnoses were depression (49.6%), smoking (41.7%), social anxiety (34.4%), suicidality (28.8%), and other abuse/addiction (26.4%). The mean number of diagnoses was 2.46 (SD 2.10). As displayed in Figure 2, most of the participants reported at least one diagnosis, whereas only 148 participants (19.2%) reported no mental disorders or substance use disorders.

**Table 5:** History of mental disorders and substance use disorders.

Social anxiety	265 (34.4%)
Panic disorder	90 (11.7%)
OCD	46 (6%)
Depression	382 (49.6%)
Bipolar disorder	31 (4%)
Psychosis/schizophrenia	13 (1.7%)
Eating disorder	79 (10.3%)
Post-traumatic stress disorder	137 (17.8%)
ADHD	174 (22.6%)
ASD	38 (4.9%)
Suicidality	222 (28.8%)
Smoking	321 (41.7%)
Alcohol abuse/addiction	117 (15.2%)
Other abuse/addiction	203 (26.4%)

OCD, obsessive–compulsive disorder; PTSD, post-traumatic stress disorder; ADHD, attention-deficit/hyperactive disorder; ASD, autism spectrum disorder.

**Figure 3:**
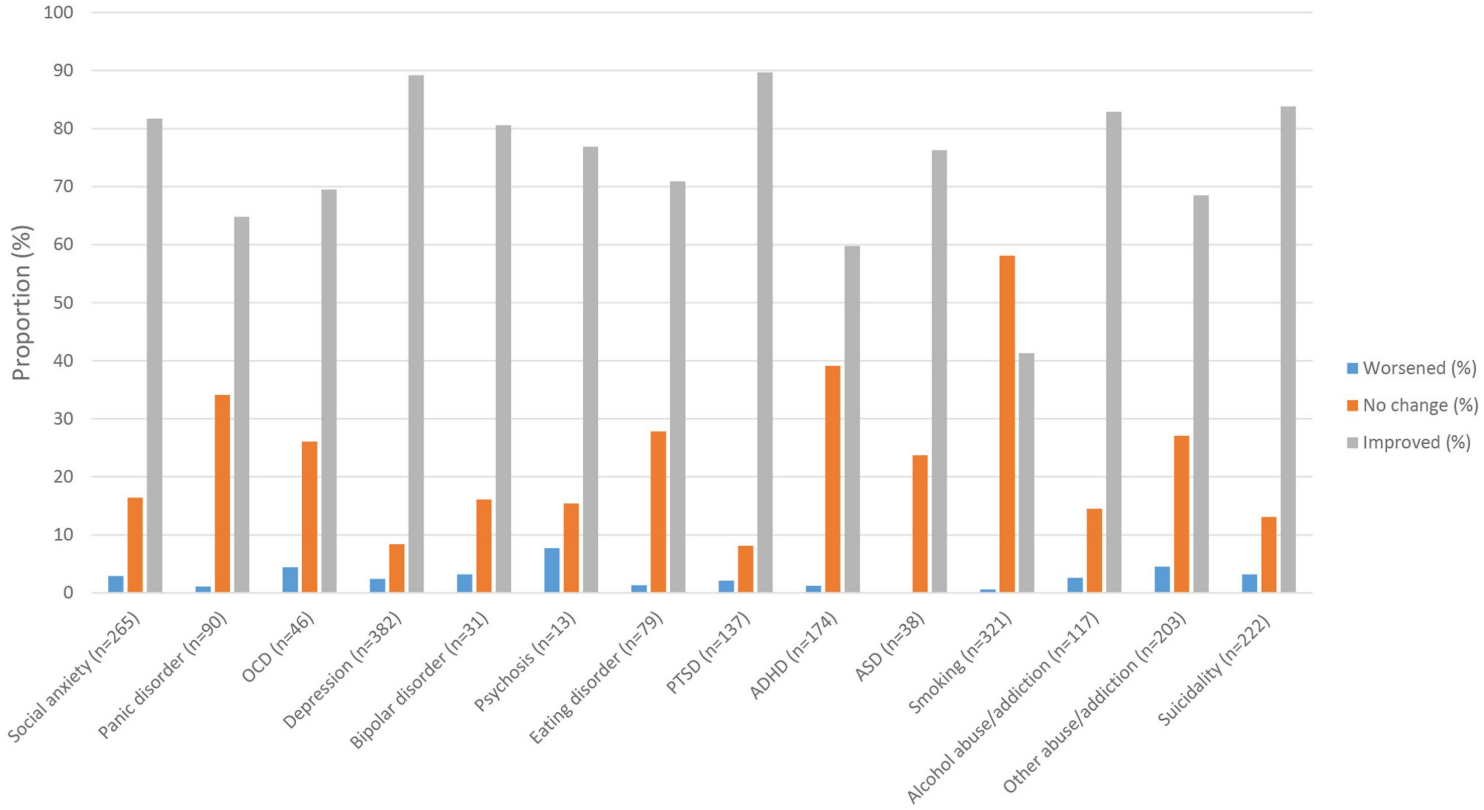
Self-perceived change in symptoms from the most memorable experience with a classic psychedelic substance. OCD, obsessive-compulsive disorder; PTSD, post-traumatic stress disorder; ADHD, attention-deficit/hyperactive disorder; ASD, autism spectrum disorder.

A small subset of participants experienced worsening of their pre-existing conditions as a result of the psychedelic experience. The conditions with the most frequently reported worsening were psychosis (7.7%), followed by other abuse/addiction (4.5%) and OCD (4.4%). Most participants reported subjectively perceived improvements in their conditions following the experience with the classic psychedelic substance. The only exception with a less than 50% improvement was smoking, of which 41.3% reported improvement due to the psychedelic experience. The conditions from which the participants reported benefit the most were PTSD (89.7%), depression (89.2%), and suicidality (83.8%). See Figure 3 for details.

### Adverse reactions

As listed in Table 6 – Persisting adverse reactions, 23.1% of the participants experienced persistent adverse reactions after the psychedelic experience. Although most of these (10.6% of the total sample) endorsed adverse reactions lasting only a few days, nevertheless a considerable proportion reported adverse reactions lasting a few weeks (4.5%) or months (3.8%), and 4.2% reported adverse reactions effects lasting even more than a year.

**Table 6:** Persisting adverse reactions, craving and unable to cut down use following the most memorable experience with a classic psychedelic substance.

**(A) Have you experienced persistent adverse reactions after the psychedelic experience?**	
No	592 (76.9%)
Yes, for a few days	82 (10.6%)
Yes, for a few weeks’ duration	35 (4.5%)
Yes, of a few months’ duration	29 (3.8%)
Yes, duration more than 1 year	32 (4.2%)
**(B) What persistent adverse reactions have you experienced? Several answer options are possible.**	
Nausea/vomiting	7 (0.9%)
Dizziness	2 (0.2%)
Anxiety/nervousness	20 (2.6%)
Paranoia	9 (1.2%)
Sadness/dejection	27 (3.5%)
Headache	14 (1.8%)
Difficult to concentrate	13 (1.7%)
Unrest/agitation	9 (1.2%)
Guilt	10 (1.3%)
Shame	8 (1%)
Irritability/anger	4 (0.5%)
**(C) Did you have to seek professional health care to handle the adverse reactions?**	
Yes	15 (1.9%)
No	412 (53.5%)
No response	343 (44.5%)
**(D) How severe were the adverse reactions?**	
Didn’t bother me at all	217 (28.2%)
Mild: did not affect function in everyday life	86 (11.2%)
Moderate: affected function in everyday life	40 (5.2%)
Severe: was unable to function in everyday life	4 (0.5%)
No response	423 (54.9%)
**(E) Have you had flashbacks/relived sensory experiences from when you were under the acute influence of the psychedelic drug some time afterwards?**	
No	579 (75.2%)
Yes, for a few days	110 (14.3%)
Yes, for a few weeks’ duration	28 (3.6%)
Yes, of a few months’ duration	31 (4%)
Yes, duration more than 1 year	22 (2.9%)
**(F) How severe were the flashbacks?**	
Didn’t bother me at all	239 (31%)
Mild: did not affect function in everyday life	70 (9.1%)
Moderate: affected function in everyday life	12 (1.6%)
Severe: was unable to function in everyday life	1 (0.1%)
No response	448 (58.2%)
**(G) After the psychedelic experience, have you experienced craving to administer the same substance again?**	
Yes	386 (50.1%)
No	384 (49.9%)
**(H) After the psychedelic experience, have you tried to cut the intake of psychedelic substances without succeeding?**	
Yes	8 (1%)
No	762 (99%)

**(B–D):** All 770 subjects were eligible; including those who did not report adverse reactions to the question in **(A)**. **(F)** All 770 subjects were eligible; including those who did not report flashbacks to the question in **(E)** Percentages are based on the total sample of 770 participants.

The most prevalent persisting adverse reactions among those who reported this (23.1%) were sadness/dejection (3.5%), anxiety/nervousness (2.6%), and headache (1.8%). Only 15 respondents (1.9%) reported that they sought professional help to handle the adverse reactions. A subset of participants reported moderate (5.2%; affected function in everyday life) or severe (0.5%; was unable to function in everyday life) adverse reactions. In order to screen for HPPD, the participants were asked about any persisting flashbacks or relived sensory experiences. Although most of the participants (75.2%) did not experience flashbacks, a considerable proportion reported symptoms of a duration of a few days (14.3%), weeks (3.6%), and months (4.0%), and 2.9% reported symptoms for more than 1 year. Most of the flashbacks were tolerated well. Only 1.6% reported affected functioning in everyday life, and only one participant was unable to function in everyday life due to the flashbacks. Half of the participants (50.1%) experienced craving to take the same psychedelic substance again, but only 1% reported failure to cut the intake of the psychedelic substance.

## Discussion

The primary aim of the present study was to study the epidemiology of classic psychedelic substances in Norway. Seventy-seven adult participants participated in an anonymous internet survey, sharing information about their most memorable experience with a classic psychedelic substance, including characteristics of the experience as well as acute and persisting effects. The sub-aims were to investigate perceived change in mental disorders and substance use disorders, and adverse reactions. Former epidemiological studies have mainly been conducted in English-speaking populations, and surveys in non-English speaking countries have been encouraged. The present study expands the limited knowledge base about the epidemiology of classic psychedelics in Scandinavia and is the first of its kind in Norway.

### Characteristics of the most memorable psychedelic experience

The study population were comprised mostly of men, 45 years or younger, unmarried persons, and with some level of higher education. Most participants reported their most memorable experience with psilocybin, followed by LSD. The motivation for the psychedelic experiences was mainly therapeutic or recreational/for fun/out of curiosity. However, these findings deviate from the outcomes of other comprehensive naturalistic and epidemiological investigations involving psilocybin, mescaline, and 5-MeO-DMT. Specifically, a naturalistic study by Mason et al. revealed that participants attending psilocybin retreats were predominantly motivated to attend this retreat to; ‘understand myself (83.6%), curiosity (80%), to resolve problems (49.1%), and other (18.2%) (26). Turning to mescaline, a prior epidemiological study highlighted that nearly all respondents were driven to consume mescaline for the purpose of exploring their spirituality or to connect with nature (16). Lastly, in the context of 5-MeO-DMT, predominant usage was linked to spiritual intentions, as indicated by studies conducted by Davis et al. (15) and Ortiz Bernal et al. (27). Most participants were well prepared for the psychedelic experience and had also done integration work afterwards. Furthermore, almost all participants reported increased understanding of past life events or relationships during the psychedelic experience.

### Perceived change in mental disorders and substance use disorders

Self-perceived or clinician-evaluated diagnoses were prevalent in the sample. Most participants with such conditions reported improvements after their most memorable experience. The findings on changes in mental disorders and substance use disorders in the present study are in line with findings from other internet surveys investigating the use of classic psychedelic substances. Like other internet surveys examining the use of 5-MeO-DMT (15), mescaline (16), DMT (17) or classic psychedelics in general (18), we found that most people experienced an improvement in their condition, and only a few percent reported a worsening. The proportion of participants who rated their most memorable psychedelic experience as top five or single most meaningful, insightful and spiritual was about the same as in the mescaline study (16), while it was lower than in two other studies (17, 18). The proportion of participants who rated the psychedelic experience as psychologically challenging was also similar to the mescaline study (16), but lower than in three other studies, two of which are on 5-MeO-DMT (13, 17, 18).

Although there has not been conducted any nation-wide assessment of the prevalence of mental disorders and substance use disorders in Norway, the lifetime prevalence was markedly higher in the sample in the present study than the lifetime prevalence in other studies, with a possible exception for bipolar, psychosis/schizophrenia and alcohol use disorder (28). The study population differs from the general Norwegian population in this regard. Nevertheless, a substantial number of participants reported improvements in their condition(s). This is in line with previous clinical research showing promising results in the treatment of depression, alcohol use disorder and other conditions (3, 5, 6). Around 40% of participants stated a therapeutic reason for the psychedelic experience. Most participants focused on preparation and integration, which are key elements of psychedelic-assisted therapy. In addition, previous life events, interpersonal relationships and coping strategies emerged during the psychedelic experience for over 90%, and most stated positive and desirable changes attributed to the experience. The participants’ adherence to treatment manuals from clinical studies with psychedelic-assisted therapy is outside the scope of the present study, but one could speculate that the modern era studies have inspired a substantial number of participants to be sensitive to set and setting to maximize the therapeutic effects and minimize challenging experiences from psychedelic substances. In line with previous research (22, 29), the participants reported their most memorable experience with a classic psychedelic drug to be rather meaningful, spiritual and psychologically insightful. In addition, they reported persisting changes in life satisfaction, social relationships, meaning of life and mood. One could assume these accounts would contribute to the perceived subjective perceived improvements in symptoms of mental disorders and substance use disorders.

### Adverse reactions

Adverse reactions were occurring in about one quarter of the participants, mostly mild and short-lived with a minimal impact on functioning, but a non-negligible number of participants reported enduring adverse reactions effects for more than a year, including persisting perceptual disturbances indicative of HPPD.

Sadness/dejection and anxiety/nervousness were the most frequently reported psychological symptoms in our material, occurring in 3.5 and 2.6% of the cases, respectively. Only 1.9% had sought professional help to handle any adverse reaction. Carbonaro et al. reported persisting depression and anxiety in 4.8 and 4.6% of the cases, respectively, more than 1 year after a challenging psychedelic experience (13), whereas 7.6% had sought professional help for the psychological symptoms. The difference between the findings in the current and the previous study can probably be explained by the fact that the latter recruited participants who had had a challenging experience under the influence of a psychedelic substance, whereas we did not include this as an inclusion criterion.

The present study sheds new light on the occurrence, duration and severity of persistent perceptual disturbances. Although we have not made a diagnosis of HPPD in the current investigation, our data will nevertheless support the assumption that HPPD is a relatively rare and mild condition that do not affect daily functioning, but that in some cases it lasts for weeks and months, and even more than 1 year.

In order to assess signals of abuse and addictive potential for the classic psychedelics in the present population, we screened for pathological patterns of behavior such as craving and unsuccessful efforts to cut down use. We found a surprisingly high rate of craving, and much higher than the 9% in a previous mescaline study (16). We asked if the participants had a desire, necessity or urge to consume the psychedelic substance again, which is a broader and more non-specific formulation than in DSM-5 (30), and may help to explain why as many as half of the participants reported craving. As evident from the present study, the motivations for use were recreational/for fun or out of curiosity in almost half of the participants, and these motivations might lead to subsequent use or abuse. Thus, the significant number of participants who wanted to take the psychedelic substance again indicates an abuse potential, but not necessarily a reinforcing effect with potential for development of a substance use disorder. The abuse potential is distinct from the addictive potential of classic psychedelic substances, which is considered to be low (8, 31). In line with this, only a small number of participants reported difficulty cutting down use.

### Limitations

The present study is subject to several limitations. First, as a cross-sectional study, it is not possible to make any causal conclusions. Second, with the retrospective design, and even though the vast majority of the participants reported that they remembered the experience clearly or very clearly, the data might be subject to recall bias. The vast majority of participants reported use of psychedelic substances on multiple occasions, and it can be hard to separate harms and benefits from the single most memorable experience from other psychedelic experiences. Third, reliance on self-reported data through the survey makes it susceptible to response bias. Some participants might have furnished socially desirable answers influenced by implicit or explicit expectations from peers, research literature, media, or other factors. Fourth, the prevalence numbers in the present study might be affected by selection bias, self-reports and the lack of confirmation from other sources. We speculate that the benefits reported by participants may have been different in a clinical trial with strict participant criteria and clinician-assessed measures. Fifth, recruitment through internet and social media advertisements might have resulted in an overrepresentation of participants with positive attitudes toward psychedelics. Those with neutral or negative attitudes may have been less inclined to participate, potentially skewing the outcomes. Notably, the survey attracted participants who had more positive attitudes toward the therapeutic effects of psychedelic substances than a previous study in a representative sample of the Norwegian population (20). Additionally, the study may have attracted participants with previous positive psychedelic experiences, whereas others with unfavorable experiences may have been more reluctant to share their experiences. Although the current study was not designed to investigate this specifically, it is conceivable that a previous psychedelic experience leads to positive attitudes toward the therapeutic use of such substances. A separate double-blind study of psychedelically naïve healthy volunteers found that 2 out of 3 ranked a psilocybin experience among the top five most meaningful experiences in life (22). Sixth, the participant sample in our survey may not be representative for the average psychedelic user, especially in terms of education, where 50.6% of the respondents had education at the university or college level. This is distinct from the general population with 36.9% reporting education at this level in 2022 (32). Therefore, our descriptive findings may have been different in a lower education sample.

Seventh, although we included 5-MeO-DMT in the survey, it is debatable whether this substance can be classified as a classic psychedelic drug. Eight, there could be other limitations, such as unknown dose and variability of effects and adverse reactions across different psychedelic substances.

Finally, we do not intend for these data to be interpreted to mean that further rigorous research is not needed. Therefore, the present observations should be replicated in not only controlled clinical trials but also naturalistic observational research to allow any strong conclusion.

## Conclusion

Despite the aforementioned limitations, this anonymous internet survey is an addition to the knowledge base about risks and benefits from psychedelic experiences in a recreational setting. In the present material, most of the participants used psilocybin or LSD, mostly for recreational and therapeutic purposes, were well prepared before the psychedelic experience and did integration work afterwards. Mental disorders and substance use disorders were prevalent in the sample, and most participants reported improvements in their condition(s). Adverse reactions were mostly tolerable and transient. However, a small subset of participants experienced persistent adverse reactions for more than a year.

## Data availability statement

The datasets presented in this article are not readily available because we used a secure web application for data storage (Services for Sensitive Data; TSD), which meets requirements for the processing and storage of sensitive research data. Access to the dataset requires special permission, and could be available on reasonable request to the first author. Requests to access the datasets should be directed to T-MK, tor-morten.kvam@so-hf.no.

## Author contributions

T-MK: Funding acquisition, Investigation, Methodology, Project administration, Visualization, Writing – original draft, Writing – review & editing. MU: Methodology, Supervision, Validation, Writing – review & editing. KA: Investigation, Methodology, Writing – review & editing. BR: Data curation, Investigation, Methodology, Writing – review & editing. PT: Data curation, Investigation, Methodology, Writing – review & editing. LS: Investigation, Methodology, Writing – review & editing. HJ: Methodology, Project administration, Supervision, Writing – review & editing. CG: Investigation, Methodology, Project administration, Supervision, Validation, Writing – review & editing.
